# Cardiovascular Associations With Monoclonal Gammopathy of Undetermined Significance: Real or Coincidental?∗

**DOI:** 10.1016/j.jaccao.2022.07.002

**Published:** 2022-09-20

**Authors:** Prashant Kapoor, S. Vincent Rajkumar

**Affiliations:** Division of Hematology, Mayo Clinic, Rochester, Minnesota, USA

**Keywords:** atrial fibrillation, cardiac, heart failure, MGUS, plasma cell

**Figure:**
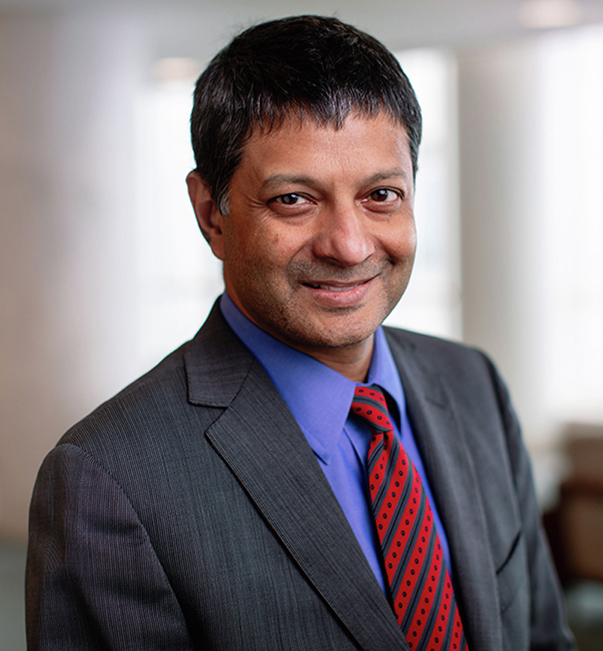

Monoclonal gammopathy of undetermined significance (MGUS) is an asymptomatic clonal plasma cell (or lymphoplasmacytic) proliferative premalignant condition characterized by the presence of a circulating monoclonal protein at a concentration of <3 g/dL, a clonal bone marrow plasmacytosis of <10%, and the absence of end-organ damage (hypercalcemia, renal insufficiency, anemia, and osteolytic bone lesions), attributable to plasma cell expansion.^1^^,^^2^ MGUS is present in approximately 5% of the general population 50 years of age and older.^3^ However, because it is an asymptomatic condition, most patients are diagnosed incidentally during the evaluation of a host of nonspecific clinical manifestations and laboratory abnormalities, including but not limited to anemia, hyperproteinemia, hypercalcemia, increased erythrocyte sedimentation rate, peripheral neuropathy, autoimmune disorders, proteinuria, and renal and skin disorders. Population-based studies have documented an age-related increase in the prevalence of MGUS, with higher rates in men compared with women, and in Black people compared with Whites.^4^^,^^5^ Genetic and shared environmental predisposition are supported by genome-wide association studies and population-based data, with an approximately 3-fold increased risk for MGUS among relatives of those diagnosed with MGUS or multiple myeloma (MM).^6^ Although MGUS invariably precedes MM, a plasma cell malignancy, the risk for progression to overt MM, immunoglobulin light-chain amyloidosis, or other related malignant lymphoproliferative disorders is small and occurs at a rate of 1% per year. In the absence of established strategies to prevent transformation to active MM, universal screening for MGUS in the general population is neither advocated nor practiced.

In a study reported in this issue of *JACC: CardioOncology*, Schwartz et al^7^ used Danish databases to evaluate the association of MGUS with cardiovascular diseases. The investigators observed a higher baseline prevalence of cardiovascular risk factors, including hypertension and type 2 diabetes mellitus, among 8,189 patients diagnosed with MGUS within the comprehensive Danish National Patient Registry between 1995 and 2018 in comparison with an age- and sex-matched control population (n = 81,890) from the same period. The setting and the underlying medical issues prompting laboratory evaluation for monoclonal proteins in which a diagnosis of MGUS was established were not captured, nor was the information regarding the tests used to establish a diagnosis of MGUS provided. With a short follow-up period (median 3.2 years for MGUS patients and 3.6 years for the control group), the study revealed a higher cumulative occurrence of cardiovascular complications among patients diagnosed with MGUS compared with the control population. The investigators assessed a gamut of cardiovascular outcomes, including heart failure, acute myocardial infarction, ischemic stroke, aortic aneurysm, aortic dissection, atrial fibrillation, valvular heart diseases, conduction disease, cor pulmonale, peripheral arterial disease, and venous thromboembolism, with multivariable-adjusted HRs exemplifying augmented risks, varying from 1.16 to 3.63, with MGUS compared with the control group. Furthermore, the study findings were unaltered in sensitivity analyses that did not consider the initial 6-month duration of follow-up after MGUS diagnosis or excluded patients with certain comorbidities including type 2 diabetes mellitus, hypertension, acute myocardial infarction, and chronic kidney diseases.

This is a large and impressive study, in which the size of the cohort permitted comparison of the risk estimates across a wide spectrum of cardiovascular outcomes. However, the study findings, akin to the results of previous studies, highlight the dilemma of whether the cardiovascular associations are real, causal, and pathogenetically related or merely coincidental. In the absence of routine screening of all patients, the difference in cardiac risks may simply reflect the underlying clinical problems that prompted patients to undergo testing for monoclonal proteins. In other words, the cardiovascular associations found may simply reflect the difference between those who underwent the testing for MGUS and those who did not, rather than the result of the test. We have previously determined that patients with MGUS may have higher mortality risk independent of progression to MM or related plasma cell disorder,^2^ and although the present study adds information on possible cardiac mechanisms, we cannot firmly conclude that a true association exists using retrospective study designs. For that, we need to use population-based screening studies in which all patients in a cohort undergo standardized screening.

Over the years, more than 130 different diseases have been reported to be associated with MGUS. Almost all of these studies have suffered from the same testing bias, making it difficult to discriminate real from coincidental associations. To solve this bias, we have in the past used data from a population-based screening study in which residents of Olmsted County 50 years of age and older were tested for the presence or absence of MGUS.^4^ We found that most of the suspected associations of MGUS are most likely coincidental.^8^

Support for the results of the Danish study come from a recent study that retrospectively screened the banked sera of a select group of subjects (n = 5,411) from the Mass General Brigham Biobank using a quantitative high-sensitivity mass spectrometry assay for detection of monoclonal protein.^9^ The available longitudinal data allowed evaluation of associations of monoclonal gammopathies with comorbidities diagnosed 6 months or later following screening of the samples, through age-adjusted logistic regression models. With a median follow-up of 4.5 years from screening, screening-detected monoclonal gammopathies correlated with increased all-cause mortality (HR: 1.55; 95% CI: 1.16-2.08; *P* = 0.0035). Interestingly, the elevated risk for overall mortality is strikingly similar to that observed in the present Danish study. Additionally, as in the Danish study, monoclonal gammopathies were associated with an increased likelihood of developing a myocardial infarction (OR: 1.75; 95% CI: 1.03-2.88; *P* = 0.039) but not ischemic stroke (OR: 0.89; 95% CI: 0.47-1.59; *P* = 0.72).

One additional caveat is that Schwartz et al^7^ included MGUS detected over 2 decades in their study. During this time, practice patterns evolved, testing for monoclonal proteins became more elaborate, newer entities, including light-chain MGUS and light-chain smoldering MM (idiopathic Bence Jones proteinuria) were defined, and myeloma-defining events were revised. Additionally, during this period, clinicians, including cardiologists, with their increased awareness of rarer conditions such as cardiac amyloidosis, likely became more vigilant, frequently examining monoclonal protein studies in appropriate settings. Although the investigators made a concerted effort to exclude patients with a known diagnosis of amyloidosis, unrecognized cases among the MGUS cohort may have also influenced the results.

To conclude, this is an interesting study that provides additional data on the possible increased cardiac risk in patients with MGUS. Data from population-based studies so far point to accelerated vascular inflammation, with consequent excess risks for venous and arterial thrombosis among people with MGUS, but the precise underlying biologic mechanisms leading to a prothrombotic state remain unclear. Elevated levels of factor VIII, von Willebrand factor, proinflammatory cytokines, platelet hyperactivation, and endothelial damage have been suggested as possible mediators. However, more data from prospective studies are needed to determine if these associations are real. The ongoing iStopMM (Iceland Screens, Treats, or Prevents Multiple Myeloma) randomized controlled trial will provide more definitive answers in this regard.^10^ Arguably, only then can we reliably consider making formal recommendations, if any, regarding the primary prevention of cardiovascular complications in patients with MGUS.

## Funding Support and Author Disclosures

This work was supported in part by grants CA168762 and CA186781 from the National Cancer Institute and by the Marvin Family Grant. Dr Rajkumar has received grants from National Institutes of Health, outside the submitted work. Dr Kapoor has received honoraria from Sanofi, Oncopeptides, Pharmacyclics, BeiGene, Cellectar, and Karyopharm, outside the submitted work.
